# 

**Published:** 1841-10

**Authors:** 


### BOOKS RECEIVED FOR REVIEW.

BRITISH.

1. Eighth Annual Report of the Trustees of the State Lunatic Hospital at Worcester (United States).—Boston, 1841. 8vo, pp. 100.

2. A Visit to thirteen Asylums for the Insane in Europe. By Pliny Earle, M.D. —Philadelphia, 1841. 8vo, pp. 144.

3. The Surgeon's Vade Mecum. By Robert Druitt. Second Edition, with 50 Wood Engravings.—London, 1841. 8vo, pp. 524. 10s. 6d.

4. A Catalogue of Plants collected in the neighbourhood of Banbury. By George Gulliver, F.R.S. F.Z.S.—London, 1841. 8vo, pp. 37.

5. A Practical Essay on some of the principal Surgical Diseases of India. By F. H. Brett, Esq. M.R.C.S.L., Bengal Med. Service.—Calcutta, 1840. 8vo, pp. 506. With 16 Plates.

6. The Retrospect of Practical Medicine and Surgery. No. III. Jan.—June, 1841. —London, 1841. 8vo, pp. 216. 4s. 6d.

7. A new Operation for the Cure of Amaurosis, Impaired Vision, and Short-sightedness. By J. J. Adams, F.L.S. G.S.—London (no date), 8vo, pp. 50. 2s. 6d.

8. Pharmaceutical Transactions. Edited by Jacob Bell. Nos. I., 8d. II., 8d. III. 1s. July—Sept. 1841. 8vo, pp. 126.

9. Observations addressed to the Chemists and Druggists of Great Britain on the Pharmaceutical Society. By Jacob Bell.—London, 1841. 8vo, pp. 15. 6d.

10. An Address delivered to the Council of the Royal College of Surgeons in London, on the occasion of quitting the Presidential Chair, July 8th, 1841. By John Painter Vincent.—Lond. 1841. 8vo, pp. 15.

11. A Letter to Sir B. C. Brodie, Bart., on the application of the Collegiate System to the Medical Schools of the Metropolis. By the Rev. J. H. North, M.A., Chaplain to St. George's Hospital.—London, 1841. 8vo, pp. 15. 1s.

12. Criminal Jurisprudence considered in relation to mental organization. By M. B. Sampson.—Lond. 1841. Roy. 8vo, pp. 29. 6d.

13. Illustrations of Phrenology. No. I. Curvoisier. By Dr. Elliottson and Mr. G. R. Lewis.—Lond. 1841. Roy. 8vo, pp. 43, 2s. 6d.

14. A Faithful Record of the Miraculous Cure of Mary Jobson. By W. R. Clanny, M.D. F.R.S. ED., Physician to the Sunderland Infirmary.—Sunderland, 1841. 12mo, pp. 64.

15. The Principles of Physiology applied to the preservation of Health, and to the improvement of physical and mental Education. By Andrew Combe, M.D., 10th Edit., revised and enlarged.—Edin. 1841. 8vo, pp. 420. 7s. 6d.

16. The Statistics of the Retreat, consisting of a Report and Tables, exhibiting the Experience of that Institution for the Insane from 1796 to 1840.—York, 1841. 8vo, pp. 100.

17. The Anatomy of the Arteries. Part VIII. By Richard Quain. 12s.

18. The Cyclopædia of Practical Surgery. Part IX.—August, 1841. 5s.

19. A new Synopsis, or the natural order of Diseases; containing their definition, principles, and treatment, with a new pathology of Fever and Inflammation. By Robert Stevens, Surgeon.—London, 1841. 8vo, pp. 175. 7s. 6d.

20. Researches into the physical History of Mankind. By J. C. Prichard, M.D. F.R.S. M.R.I.A. 3d Edit. Vol. III. Part I. —London, 1841. 8vo, pp. 507. 16s.

21. Queries respecting the Human Race, to be addressed to Travellers and others. Drawn up by a Committee of the British Association.—London, 1841. 8vo, pp. 14.

22. Outlines of a Course of Lectures on Medical Jurisprudence. By Professor Traill, of Edinburgh. Revised, with Notes, by Professor Dunglison.—Philadelphia, 1841. 8vo, pp. 234.

23. Popular Lectures on Man; his Structure and Functions, considered with reference to Health, Culture, and Natural Theology. Delivered at the Highgate Institution. By John White, Surgeon.—London, 1841. 8vo, pp. 252. 5s.

24. On the Construction and Management of Hospitals for the Insane. By Dr. M. Jacobi. Translated by J. Kitching. With an Introduction by S. Tuke.—Lond. 1841. 8vo, pp. 299. 9s.

25. Observations on the Structure and Diseases of the Testis. By Sir Astley Cooper, Bart., F.R.S. Second Edition, Edited by Bransby Cooper, F.R.S.—Lond. 1841. 4to, pp. 320. With Plates. 3*l*. 3s.

26. Three Memoirs on the Development and Structure of the Teeth and Epithelium; read at the Ninth Annual Meeting of the British Association held at Birmingham in August, 1839. By A. Nasmyth, F.L.S. F.G.S., Member of the Royal College of Surgeons.—London, 1841. 8vo, pp. 47, with many Plates. 5s.

27. The Graveyards of London; being an exposition of the physical and moral consequences of depositing the dead in the midst of the living. 1841. 8vo, pp. 32. 1s.

28. The Cure of Strabismus by Surgical Operation. By W. P. Mackenzie, M.D., &c.—London, 1841. 8vo, pp. 30.

29. A Practical Treatise on the Diseases of Children. By James Stewart, M.D.—New York, 1841.—8vo, pp. 547. 18s.

30. State of the Lincoln Lunatic Asylum. 1841.—Lincoln. 8vo, pp. 79.

31. Tic Douloureux, or Neuralgia Facialis, and other Nervous Affections; their seat, nature, and cause; with cases illustrating successful methods of treatment. By R. H. Allnatt, M.D. A.M.—London, 1841. 8vo, pp. 184.

32. A Practical Treatise on the Efficacy of Mineral Waters in the Cure of Chronic Disease. By Sir Alexander Mackenzie Downie, M.D.—Frankfort, 1841. 12mo, pp. 219. 6s.

33. A Short Description of Kissingen; its Baths and Mineral Waters. Translated from the German of Dr. F. A. Balling. By Sir Alex. M. Downie, M.D.—Frankfort, 1841. 8vo, pp. 44. 1s. 6d.

34. Diseases of the Ear and Hearing; together with Ectropium. By T. Wharton Jones, F.R.S., Lecturer on Anatomy and Physiology at Charing Cross Hospital Medical School. From the Cyclopædia of Practical Surgery.—London, 1841. Royal 8vo, pp. 64.

35. The Oration delivered before the Medical Society of London at their Anniversary, March 8, 1841.—By W. D. Chowne, M.D. Printed at the Society's request.—London, 1841. 8vo, pp. 71.

36. Report of the Poor Law Commissioners of Medical Charities in Ireland.—Dublin, 1841. 8vo, pp. 167.

37. Case of a Man who passed fourteen large intestinal Concretions; with remarks. By R. Turner, Surgeon; and an account of the constitution of the concretions; with notices of similar concretions. By Douglas Maclagan, M.D. Extracted from the Edinb. Monthly Journ.—Edinb. 1841. 8vo, pp. 28.

38. Thoughts on Quarantine and other Sanatory Systems; being an Essay which received the Boylston Prize in 1834. By Charles Caldwell, M.D.—Boston, 1834. 8vo, pp. 72.

LIBRARY OF THE

Orleans Parish Medical Society

### INDEX TO VOL. XII.

OF THE

### BRITISH AND FOREIGN MEDICAL REVIEW.

| | PAGE |
|---|---|
| Abortion, remarkable case of . | 431 |
| medico-legal case of . | 562 |
| Abscess of the liver, treatment of | 89 |
| mammary, compression in | 259 |
| Acton, Mr. on syphilis . . | 362 |
| Adhesions of pericardium, age of | 134 |
| Albumen, identity of with fibrin | 563 |
| Almanac, medical, American . | 223 |
| Amenorrhœa treated by electricity | 345 |
| Amputation, statistics of . . | 260 |
| Amesbury, Mr. on spinal curvature | 177 |
| Anatomical character of diseases . | 98 |
| Anatomy, comparative, Mr. Jones on | 218 |
| Anderson, Dr. on typhus fever . | 294 |
| Aneurism, Mr. Porter on . | 438 |
| varieties of . . | 443 |
| Angina, simple, contagious . | 537 |
| Anthelmintics, Abyssinian . | 250 |
| Anus, artificial . . . | 460 |
| supplementary muscles of | 331 |
| Aorta, transposition of . . | 332 |
| Aphthæ orientales . . | 516 |
| Arnott, Dr. J. on stricture . | 388 |
| Arsenic, use of, in phthisis . | 249 |
| Mr. Taylor on . . | 342 |
| not naturally in bones . | 347 |
| poisoning by . . | 564 |
| Arteries, diseases of . . | 112 |
| morbid closure of . | 134 |
| wounds of . . | 441 |
| Ashwell, Dr. on undue lactation . | 328 |
| Asphyxia, Dr. Carpenter on . | 112 |
| from carbonic acid . | 264 |
| Dr. Reid on . . | 278 |
| Authority in medicine, its merits | 53 |
| Azores, Dr. Bullar's account of | 224 |
| climate of . . | 172 |
| Babington, Dr. on epilepsy . | 340 |
| Bahamas, climate of . . | 172 |
| Bandaging, new system of . | 252 |
| Barlow, Dr. on diabetes . . | 334 |
| on the laws of tubercles | 348 |
| Barry, Dr. on the chorda dorsalis | 570 |
| on the blood corpuscles | ib. |
| Bayard, M. on medical jurisprudence | 174 |
| Baumès, M. on syphilis . . | 362 |
| Belladonna in paraphimosis . | 556 |
| Bermuda, climate of . . | 172 |
| Bile, non-elimination of . . | 280 |
| duct, structure of . . | 133 |

| | PAGE |
|---|---|
| Bingham, Mr. on the insane . | 54 |
| Bird, Dr. on a new test of pregnancy | 326 |
| on electricity as a remedy | 344 |
| Blake, Mr. on the blood . . | 571 |
| Blenorrhagia in the female . | 365 |
| Blindness, singular case of . | 567 |
| Blood, experiments on . . | 147 |
| analysis of morbid . | 270 |
| Mr. Blake on . . | 571 |
| corpuscles, Dr. Barry on . | 570 |
| Bones, on the diseases of . . | 154 |
| inflammation of . . | 155 |
| caries and necrosis of . | 156 |
| mollities of . . | 158 |
| Bonnet, M. on diseases of the liver | 78 |
| Books received for review | 291, 577 |
| Brain, inflammation of . . | 100 |
| influenced by pericardium . | 333 |
| chemistry of . . | 563 |
| Brett, Mr. on Indian surgery . | 422 |
| Brodie, Sir B. on local nervous affections . . . | 121 |
| Bronchitis of children, works on . | 350 |
| Bronchocele, Dr. Rowland on . | 119 |
| Bronchotomy, observations on . | 110 |
| remarkable case of | 256 |
| Bullar, Dr. on the Azores . | 224 |
| Calculus, effects of medicines in . | 390 |
| injections in . | 398 |
| removal of by dilatation | 400 |
| its prevalence in India . | 425 |
| treatises on . . | 388 |
| Carbonic acid, asphyxia from . | 264 |
| Cartilages of the bronchi . | 332 |
| Cephalalgia, Dr. Bennett on . | 104 |
| Children, dyspepsia of . . | 169 |
| cerebral affections of . | 346 |
| asphyxiated, treatment of | 560 |
| treatment of fractures in | 544 |
| China, establishment of hospitals in | 290 |
| Chorda dorsalis, Dr. Barry on . | 570 |
| Chorea, Dr. Thompson on . | 106 |
| use of spirits in . . | 284 |
| Christison, Dr. on tyyhus fever . | 293 |
| Civiale, M. on calculus . . | 388 |
| on contraction of urethra | 557 |
| Claessen, Dr. on the cold water cure | 189 |
| Clark, Sir James, on climate . | 160 |
| Clavicle, backward dislocation of | 551 |
| Climate, Sir James Clark on . | 160 |

PAGE
Climates, English, account of . 168
Combe, Dr. on health in infancy . 228
his Physiology . 513
Compression in white swelling . 512
Consumption, choice of a climate for 162
Contractility, muscular, Dr. Reid on 277
Cooper, Mr. B. on hernia . 332
Sir A. on the testis . 515
Cornea, iodine in opacity of . 258
Croup, Dr. Williams on . . 109
cure of . . . . 246
Cruveilhier, M. on the sounds of the heart . . . . . 533
Cryptogamia in animals . . 557
Cupping, dry, new mode of . 183
Curvature of the spine, treatises on 177
spinal, section of muscles in 283
Davis, Dr. his Midwifery . 461
Davy, Dr. his Researches . 129
Delaroque, M. on typhus fever . 293
Delirium tremens . . 103
Dieffenbach, M. on stammering . 209
Dislocation of the shoulder . 454
of clavicle backward . 551
Donné, M. on the urine . . 539
Donovan, Mr. on vinum ferri . 568
on quinina . . 569
Diabetes, Dr. Barlow on . . 333
Dracunculus in India . . 429
Dropsy of the pericardium . 281
Dyspepsia, its relation to mind . 276
Ecchymosis in injuries of head . 550
Edinburgh, typhus fever of . 293
Ectropium cured by operation . 252
Elbow-joint, excision of . 253, 330
Electric currents in nerves . 245
Electricity as a remedy . . 344
of the torpedo . . 129
Elephantiasis in India . . 424
Empyema, new operation for . 251
Enema, tobacco, poisoning from . 562
Entomological Society, transact. of 219
Epilepsy, Dr. Babington on . 340
ligature of arteries in . 556
Eye, section of muscles of, in the horse . . . . . 251
Eye, on the aponeuroses of . 258
galvanism in diseases of . 542
hairs within the . . 549
on the actions of . . 553
Eyelids, horny excrescence on . 248
Femur, congenital dislocation of . 259
Fever, typhus, treatise on . 294
intermittent, Dr. Shapter on 93
transmitted . 246
puerperal, in Paris . 536
Fibrin and albumen, identity of . 563
Forget, M. on typhus . . 294
Foundling Hospital of St. Petersburg 273
Fracture, ununited, seton in . 255
France, climates of . . 169
Franz, Dr. his cure of blindness . 567
Galvanism in diseases of the eye . 542

PAGE
Gastritis, Dr. Symonds on . 113
Gastrotomy in the cow . . 253
Gavarret, M. on statistical medicine 1
Gerhard, Dr. on typhus . . 293
Gibson, Dr. his Rambles in Europe 232
Glasgow, typhus fever in . . 294
Goodlad, Mr. on nervous affections 121
Gout, Mr. Parkin on . . 507
Graves, Dr. sketch of . . 233
Gravity, specific, of organs . 134
Griffin, Dr. on abdominal inflammation 280
Gruithuisen, Dr. on solution of the stone . . . . 405
Guerin, M. on spinal curvature . 177
Guinea-worm in India . . 429
Guy's Hospital Reports . . 326
Hall, Dr. on the nervous system . 200
Hanging, signs of . . 270
curious case of . . 560
Health, Dr. Combe on preserving 513
Heart, Dr. Joy on diseases of . 112
motions or sounds of . 533
Heat, animal, Dr. Davy on . 139
Hectic fever, Dr. Christison on . 95
Hemeralopia, epidemic . . 538
Hemorrhage, uterine, cold water in 261
Hemorrhois . . . 117
Henderson, Dr. on typhus fever . 293
Hermaphrodite, history of . 332
Hernia, new variety of . . 546
of the uterus . . 261
inguinal, anatomy of . 511
radical cure of . . 332
Hodgkin, Dr. on typhus fever . 294
Holst, Dr. on the health of prisoners 498
Hope, Dr. character of . . 286
Horses, venereal disease of . 494
Hughes, Dr. on incipient phthisis 331
on hydrocephalus . 346
Humerus, excision of . . 545
Hydrocele, M. Velpeau on . 452
Hydrocephalus, Dr. Bennett on . 103
caused by tubercles 229
Dr. Vrolik on . 228
Dr. Hughes on . 546
cold affusion in . 559
Hysteria, Dr. Laycock on . 60
Dr. Thompson on . 106
Ileum, separation of part of . 540
Ileus from diseased pancreas . 538
India, surgical diseases of . 422
Infancy, Dr. Combe on . . 228
Infantile fever, Dr. Locock on . 95
Inflammation, abdominal, diagnosis of 280
Influenza, Dr. Thompson on . 111
Infusoria, circulation of . . 245
Injections, vaginal and uterine 215, 263
Insane, their religious delusions . 54
Insanity, Dr. Prichard on . 103
Insects within the body . . 219
Iodine, tincture of, in chancre . 251
Italy, climates of . . . 170
Itch, treatment of, at Berlin . 254

31

| | PAGE |
|---|---|
| Jurisprudence, on the study of . | 194 |
| Key, Mr. on excision of the elbow | 330 |
| Kiestein, a test of pregnancy | 326, 566 |
| Lactation, on undue . . | 328 |
| anomalous . . | 431 |
| Laryngotomy . . . | 260 |
| Lavacherie on compression . | 512 |
| Laycock on nervous diseases . | 60 |
| Lead, poisoning by . . | 249 |
| Lee, Mr. on stammering . . | 209 |
| Leeches, poisoning by . . | 247 |
| Leg, new artificial . . | 541 |
| Leroy, M. on calculus . . | 388 |
| Lever, Mr. his obstetrical statistics | 343 |
| Library of Medicine, Dr. Tweedie's | 92 |
| Lisfranc, M. sketch of . . | 233 |
| Lithotomy, M. Souberbielle on . | 194 |
| at Naples . . | 254 |
| Lithotrity, origin of . . | 407 |
| estimate of . . | 415 |
| Liver, diseases of, treatises on . | 78 |
| London, climate of . . | 166 |
| Louis, M. on typhus fever . | 22, 293 |
| Leucorrhœa, treatment of . . | 459 |
| Mr. Brett on . | 427 |
| Lying-in Charity, Guy's . . | 343 |
| Mackenzie, Dr. on vision . | 217 |
| Madeira, climate of . . | 171 |
| Malta, climate of . . . | ib. |
| Mandl, M. on the blood . . | 270 |
| Medicine, principles of . . | 1 |
| Mesenteric glands, disease of . | 117 |
| Metropolitan Convalescent Institution | 576 |
| Midwifery, treatises on . . | 461 |
| Milk, microscopical characters of | 263 |
| elements of, in urine . | 326 |
| abnormal secretion of . | 558 |
| Mind, its relation to dyspepsia . | 274 |
| Monsters, Dr. Vrolik on . . | 374 |
| Mortality, London tables of | 285, 575 |
| Morton, Mr. on hernia . . | 511 |
| Muscles of the hand, division of . | 257 |
| spine, division of . | 181 |
| Myopia, cure of, by operation . | 553 |
| Nails, condition of, in fracture . | 548 |
| Nasal ducts, absence of . . | 541 |
| Nasmyth, Mr. on the teeth . | 491 |
| Nerves, anastomosis of . . | 236 |
| functions of the roots of . | 239 |
| cerebral, motor influence of | ib. |
| Nervous diseases, Dr. Laycock on | 60 |
| local . . | 121 |
| system, Dr. Bennett on . | 99 |
| Dr. Hall on . | 200 |
| Neuralgia cured by turpentine . | 432 |
| operation . | 283 |
| Nitre in rheumatism . . | 247 |
| Numerical method, the . . | 1 |
| Operations in America, statistics of | 223 |
| Orchitis, new treatment of . | 256 |
| Osiander on uterine tubercles . | 558 |
| Oxalic acid, its medical use . | 539 |
| Ozæna, on the cure of . . | 549 |
| Pancreas, fatal hypertrophy of . | 538 |
| Paralysis treated by electricity . | 345 |
| Paraphimosis, use of belladonna in | 556 |
| Pedicular disease . . . | 432 |
| Perception, suspended case of . | 565 |
| Pericarditis, effect on the brain of | 333 |
| Pericardium, sanguineous effusion in | 250 |
| dropsy of . . | 281 |
| Petit, M. on the Vichy water . | 588 |
| Petersburg, Foundling Hospital of | 273 |
| Pharmacopœia, the Prescriber's . | 221 |
| Philadelphia, typhus of . . | 294 |
| Phillips, M. on stammering . | 209 |
| Philosophers, non-philosophy of . | 208 |
| Phlebitis, diagnosis of . . | 431 |
| Phlegmasia dolens . . | 112 |
| Phthisis, arsenic used in . . | 249 |
| iodine in . . | 282 |
| treatment of incipient . | 331 |
| Plague, Dr. Shapter on . . | 92 |
| Plica Polonica, pathology of . | 247 |
| Pneumonia of children . . | 350 |
| Pneumothorax, treatment of . | 257 |
| traumatic . | 552 |
| Poison of animals . . | 157 |
| Porter, Mr. on aneurism . . | 438 |
| Parkin, Mr. on gout . . | 507 |
| Prisons, health of . . | 498 |
| Provincial Association, trans. of | 231 |
| Pruritus scroti, case of . . | 548 |
| Puncture in liver abscess . . | 85 |
| Pus, observations on . . | 135 |
| Pyrosis, Dr. West on . . | 503 |
| Quinine, mode of prescribing . | 569 |
| Rambles in Europe . . | 232 |
| Ramsbotham, Dr. his Midwifery . | 461 |
| Rectum, scirrhus of . . | 256 |
| Rees, Dr. on the urine . . | 345 |
| Reid, Dr. on typhus fever . | 293 |
| on contractility . | 276 |
| on asphyxia . . | 278 |
| Religion in relation to insanity . | 54 |
| Rheumatism treated by nitre . | 247 |
| Richter on diseases of bones . | 154 |
| Rickets, M. Guerin on . . | 187 |
| Riga, medical trans. of . . | 430 |
| Rigby, Dr. his Midwifery . | 461 |
| Robertson, Dr. on the spine . | 177 |
| Roods, Mr. on the spine . . | 177 |
| Royal College of Surgeons . | 576 |
| Science, natural, cyclopædia of . | 227 |
| Scorbutus, pectoral effusions in . | 250 |
| Scrofula, walnut-leaves in . | 539 |
| Secale cornutum, essay on . | 509 |
| Seton in ununited fracture . | 255 |
| Silver, chloride of . . | 567 |
| Skey, Mr. on the spine . . | 177 |
| Skull, on the repair of . . | 226 |
| Smallpox, Dr. Gregory on . | 95 |
| Smith, Dr. on yellow fever . | 433 |
| Souberbielle, M. on lithotomy . | 194 |
| Snakes, on the poison of . . | 137 |
| Serney, Dr. on the spine . . | 177 |

PAGE

Spina bifida, radical cure of . 546

Spinal curvature, treatises on . 177

cord, on the functions of 235, 245

marrow, treatise on . 514

pathology of . 535

Stammering, dangerous operation in 252

first operation for . 257

Statistical medicine, Gavarret on . 1

Stewart, Dr. on typhus fever . 294

Stomatitis, Dr. Symonds on . 113

Stone, high operation for . . 194

treatises on . . 388

Strabismus cured without operation 546

origin of operation for 564

operation for . . 554

Stricture of the colon . . 116

new method of treating . 388

Strangulation, intestinal, internal 538, 551

Stuttering, on the cure of . 209

Suicide, anatomy of . . 149

Surgeons, London College of . 576

Sutures of caoutchouc . . 282

Syphilis in pregnant women . 541

treatises on . . 362

primary, Mr. Key on . 335

Teeth, on the development of . 491

Temperature of animals . . 139

Testis, Sir A. Cooper on . . 515

Thomson, Dr. on the liver . 78

Tissues, non-vascular . . 572

Toad, on the poison of . . 137

Tobacco enema, poisoning from . 562

Tongue, incision of, in stammering 210

Tonsils, removal of, in stammering 213

Torpedo, experiments on . . 129

Taylor, Mr. on arsenic . . 342

Toynbee, Mr. on non-vascularity 572

Tubercles, cause of hydrocephalus 229

Dr. Barlow on . 348

in the uterus impeding labour . . . . . 558

Tuson, Mr. on spinal curvature . 117

Typhus fever, treatises on . 294

identity of, in France and England . . . 293

abdominalis . . ib.

PAGE
Urethra, contraction of . . 557
Urethroplasty, new . . 543
Urine, lead found in . . 249
antimony found in . . ib.
composition of, in pregnancy 539
Uterine hemorrhage . . 118
Uterus, hernia of . . . 261
retroversion of . . ib.
foreign bodies in . . 262
congestion of . . 263
Dr. Rees on . . 345
a test of pregnancy . 326
wanting . . . 540
Valleix, M. on typhus . . 293
Van Deen on the spinal marrow . 514
Variable diseases . . . 331
Varicocele . . . 457
Variola after vaccination . . 430
Varix . . . . 456
Veins, on air in . . . ib.
diseases of . . . 112
Velpeau, M. his Lectures . 452
Venereal diseases, Mr. Acton on . 362
disease of horses . 494
Vichy, waters of, in stone . 388
Vidal, M. on uterine injections . 215
Vinum ferri, preparation of . 568
Vision, physiology of . . 217
improved by myotomy . 253
Volkmann, M. on the nerves . 239
Volz, Dr. his medical facts . 254
Vrolik, Dr. on repair of the skull 226
on hydrocephalus . 228
on double monsters 374
Walnut-leaves in scrofula . 539
Ward, Mr. on the spine . . 177
Wardleworth, Mr. on secale . 509
Water, new system of cure by . 189
West, Dr. on pyrosis . . 503
Indies, climate of . . 172
White swelling, new mode of treating 550
Winslow, Mr. on suicide . . 149
Wry neck, M. Guerin on . 187
Yearsley, Mr. on stammering . 209
Yellow fever, Dr. Shapter on . 94
Dr. Smith on . 433

END OF VOL. XII.

C. Adlard, Printer, Bartholomew Close.

